# Direct laser induced writing of high precision gold nanosphere SERS patterns

**DOI:** 10.1039/d3na00855j

**Published:** 2024-02-01

**Authors:** Olympia Geladari, Philipp Haizmann, Andre Maier, Markus Strienz, Martin Eberle, Marcus Scheele, Heiko Peisert, Andreas Schnepf, Thomas Chassé, Kai Braun, Alfred J. Meixner

**Affiliations:** a Institut für Physikalische und Theoretische Chemie, Universität Tübingen Auf der Morgenstelle 18 D-72076 Tübingen Germany alfred.meixner@uni-tuebingen.de kai.braun@uni-tuebingen.de; b Center for Light-Matter Interaction, Sensors & Analytics LISA^+^, Universität Tübingen Auf der Morgenstelle 15 D-72076 Tübingen Germany; c Institut für Anorganische Chemie, Universität Tübingen Auf der Morgenstelle 18 D-72076 Tübingen Germany andreas.schnepf@uni-tuebingen.de

## Abstract

The high sensitivity and molecular fingerprint capability of Surface-Enhanced Raman Spectroscopy (SERS) have lead to a wide variety of applications ranging from classical physics, chemistry over biology to medicine. Equally, there are numerous methods to fabricate samples owing to the desired properties and to create the localized surface plasmon resonances (LSPRS). However, for many applications the LSPRs must be specifically localized on micrometer sized areas and multiple steps of lithography are needed to achieve the desired substrates. Here we present a fast and reliable direct laser induced writing (DIW) method to produce SERS substrates with active areas of interest in any desired size and shape in the micrometer regime. Afterwards, the SERS substrates have been functionalized with phthalocyanines. The DIW fabricated samples realize sub-monolayer sensitivity and an almost uniform enhancement over the entire area, which make this production method suitable for many sensing applications.

## Abstract 2

Introducing a fast, reliable, and flexible “Direct Laser Writing” technology for printing precise and complex SERS-patters on different substrates of any desired structure with nanometer resolution for applications in sensor technology and optoelectronics.

## Graphical abstract

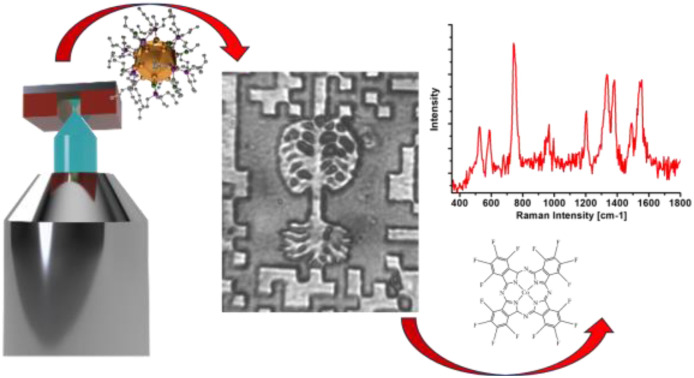

## Introduction

Noble-metal nanoparticles (NPs) have aroused widespread research interest due to their unique physical and chemical properties compared to their bulk counterparts, with one of the most fascinating aspects being their optical properties.^1^ These NPs exhibit strong absorption in the visible regime of the spectrum, which is attributed to collective electron oscillations in the conduction band, known as surface plasmons, in response to the electric field of the incoming light.^2–4^ The prevalent spectroscopic technique that benefits from this phenomenon is Surface-Enhanced Raman scattering (SERS), which has attracted considerable attention since its discovery in 1974.^5–7^ SERS uses the electromagnetic field scattered at metal NPs or roughened metallic surfaces to greatly amplify molecule-specific Raman signals, representing a powerful analytical tool for ultrasensitive, non-destructive and real-time detection.^8^ Signal amplification by many orders of magnitude becomes accessible and has been widely applied in the fields of sensing,^9,10^ molecular electronics^11^ and single molecule spectroscopy.^12–17^ Moreover SERS can be combined with other advanced techniques, such as *in vivo* imaging^18–22^ and microfluidics.^23–28^ Despite the rapid development in this field, the generation of reproducible and applicable SERS substrates turns out to be a challenging task, that ranges from chemical procedures harnessing colloidal metallic solutions^29–32^ and reverse micelles,^33,34^ to methods like Nanosphere lithography (NSL),^35^ Nanoimprint lithography (NIL)^36^ and Electron-beam lithography (EBL).^37,38^ Many of the substrates made in these ways are limited due to their air sensitivity and general instability (*e.g.* those based on silver), and their fabrication requires experience in nanoparticle synthesis or needs highly specialized equipment.^39^ Inhomogeneities in the size and shape of the NPs, because of varying preparation recipes, can lead to inconsistency and unreliable SERS performance. In the case of EBL-fabrication the disadvantages are evident: the time for pixel-by-pixel scans is long with low throughput, while system maintenance can be very expensive and extensive. In addition, subsequent processing steps like metal lift-off and etching, can be troublesome at the nanoscale.^40^ To overcome these obstacles, the aim is to develop a simple and adaptable approach for the implementation of well-characterized NPs on a variety of surfaces.

Several promising alternative techniques have been introduced recently for producing uniform, large scale high sensitive SERS substrates such as superelastic nanomolding of sub-micrometer metallic pillar arrays,^41,42^ optical nanoprinting of colloidal particles by optical gradient and scattering forces on a large variety of different substrates and the formation of light-induced solid-state protrusion of gold nanowires^43^ or the integration of robust SERS substrates by direct induced laser writing (DIW) with femtosecond laser pulses.^44–46^

In the present work, we report a new time-saving and cost-effective method for the fabrication of SERS substrates, that makes use of the local surface plasmon resonance (LSPR) of gold nanospheres (Au-NS) with an average size of about 20 nm. The main advantages of this new technique are the fast writing speeds with up to 100 μm s^−1^, the low cw-laser power that can be used for excitation instead of high-power femtosecond laser pulses for sensitive applications. Most importantly we use gold instead of silver as the targeted material in contrast to most other DIW methods introduced before. The Au-NS are deposited in a targeted manner onto the surface of Si/SiO_*x*_ wafers by laser-induced printing, allowing us to control the positioning of the nanosphere arrangement with high precision. For adjacent Raman spectroscopy investigations, we fabricate a proof-of-concept SERS substrate, using a transition-metal phthalocyanine (TMPc) as probe molecule to study the SERS performance of our printed Au-NS structures.

## Results

### Principle of the Au-NS SERS substrate generation

**Fig. 1:**
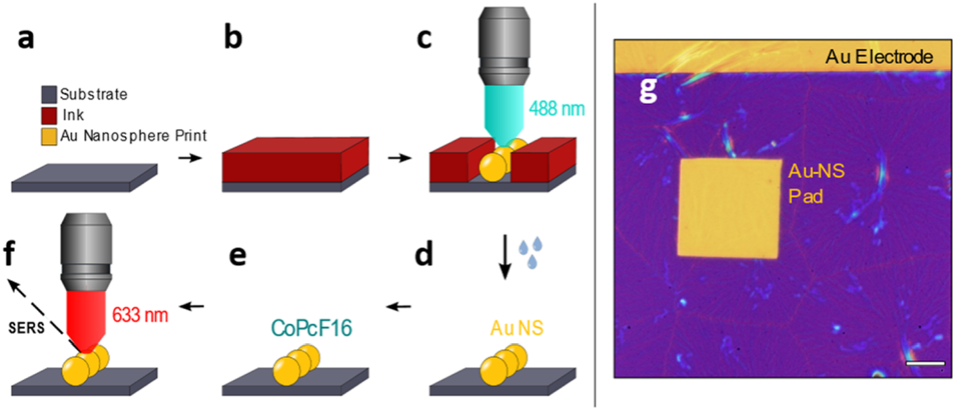
Laser-induced printing of Au nanospheres on a surface of choice. (a–f) Scheme for printing of a ready-to-measure SERS substrate. (a and b) Spin-coating of a thin film of the ink solution onto the substrate surface. (c) Selective printing of an Au-NS structure by raster scanning the sample through the focused laser beam, causing the Au_32_ nanocluster to coalesce to Au-NS. (d) Lift-off: wet removal of the unexposed ink film leaves a clean surface with the Au-NS pattern on top. (e) Uniform thin layer deposition of CoPcF_16_ on the substrate. (f) Detecting SERS of the CoPcF_16_ molecule on top of the Au-NS pattern. (g) Optical micrograph of a printed rectangular Au-NS pad (scale bar 10 μm).

Recently, we have developed a direct printing method to create gold electrodes on arbitrary samples.^47,48^ We have adopted this method and expand it to produce samples with desired nano structures, which can be used for SERS spectroscopy. The ink solution consists of Au_32_(^*n*^Bu_3_P)_12_Cl_8_ nanoclusters (Au_32_),^49^ which will locally form structures of gold nanospheres (Au-NS), through selective exposure to focused laser radiation. The printing process is sketched in Fig. 1 and works as follows.

A 0.5 mM concentrated solution of the Au_32_ nanoclusters (in hexane) is spin-coated onto a substrate of interest *e.g.* a Si/SiO_*x*_ piece of a wafer to form a closed film (Fig. 1a and b). The writing is then performed on a home-built inverted confocal microscope, which is equipped with a 488 nm cw-laser (Toptica) and controlled by a pattern generator (HydraSpex) that synchronizes sample scanning with selective spot by spot laser illumination on the sample surface. Upon illumination the Au_32_ nanoclusters in the radiation sensitive ink are absorbing light and convert into elemental gold, which starts to agglomerate and forming bigger particles, mostly nanospheres. The sizes and distances of this single gold nanospheres (Au-NS) depend on thickness and concentration of the film and on the variable duration of the illumination (Fig. 1c). This leads to the arrangement of the Au-NS in a predefined manner with nm-precise positioning. In a following lift-off step (Fig. 1d) the unexposed thin film can be removed resulting in a clean wafer with the remaining Au-NS pattern. Afterwards a thin layer of perfluorinated cobalt phthalocyanine (CoPcF_16_) is deposited onto the Au-NS patterned silicon substrate (Fig. 1e). We choose CoPcF_16_ to trace the fluor signal in X-ray photoelectron spectroscopy (XPS) measurements. The molecules will act as Raman scatterers to determine the optical properties of our proof-of-concept system for potential surface enhanced Raman scattering investigations (SERS, Fig. 1f). This samples are prepared on commercial finger electrode substrates, which work as recognition structure and allow fast orientation on the sample. Fig. 1g shows the optical micrograph of a rectangular printed pad consisting of gold nanospheres (Au-NS-pad), that was printed underneath a pre-existing gold electrode. The thin ink film is yet to be removed.

### Optical and structural characterization of the SERS substrate

**Fig. 2:**
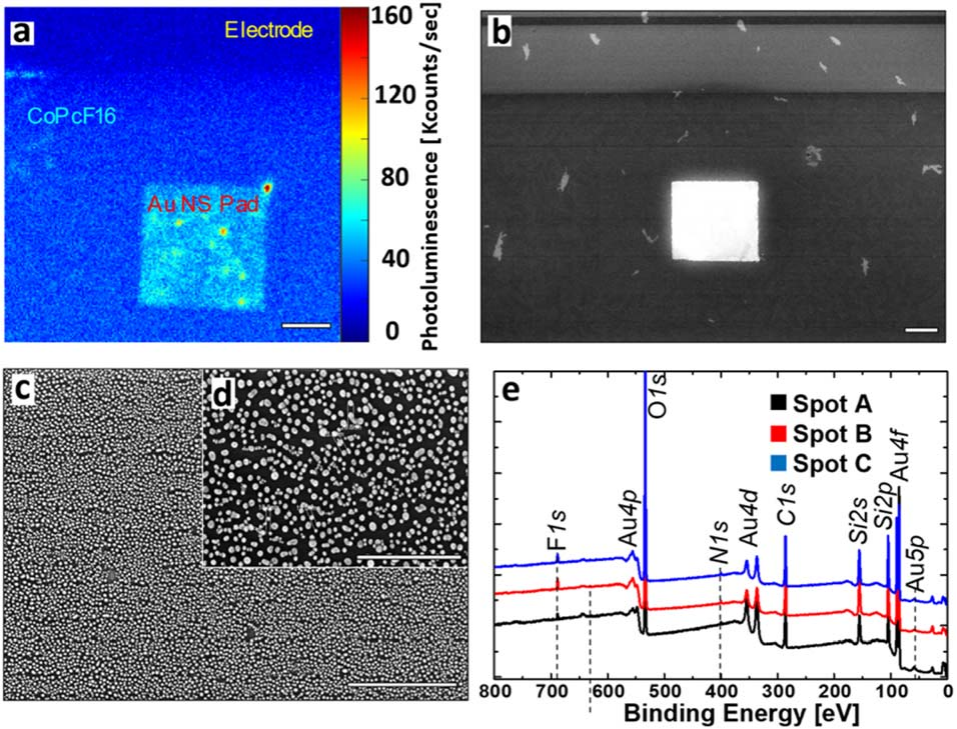
Characterization of the fabricated proof-of-concept SERS substrate. (a) Photoluminescence image of the Au-NS pad and surroundings at excitation with 633 nm. Mainly the PL of the CoPcF_16_ molecule is observed, which is enhanced on the Au-NS surface. Scale bar: 10 μm. (b) Scanning electron micrograph of the Au-NS pad and surroundings. Scale bar: 10 μm. (c) Zoom into the Au-NS pad revealing its nanosphere constitution. Scale bar: 1 μm. (d) High resolution SEM image. The average nanosphere size is determined as 20.5 ± 7.2 nm with maximal grain distances of ≈50 nm. Scale bar: 500 nm. (e) XPS spectra confirming the presence of a thin CoPcF_16_ layer on the substrate surface with three probe spots (A–C).

To characterize the nano structure of our laser written Au structures we investigated their optical and structural properties by confocal and scanning electron microscopy (SEM). Additionally, we performed X-ray photoelectron spectroscopy, to verify the uniform presence of a sub-monolayer of CoPcF_16_. The results are displayed in Fig. 2.

**Fig. 3:**
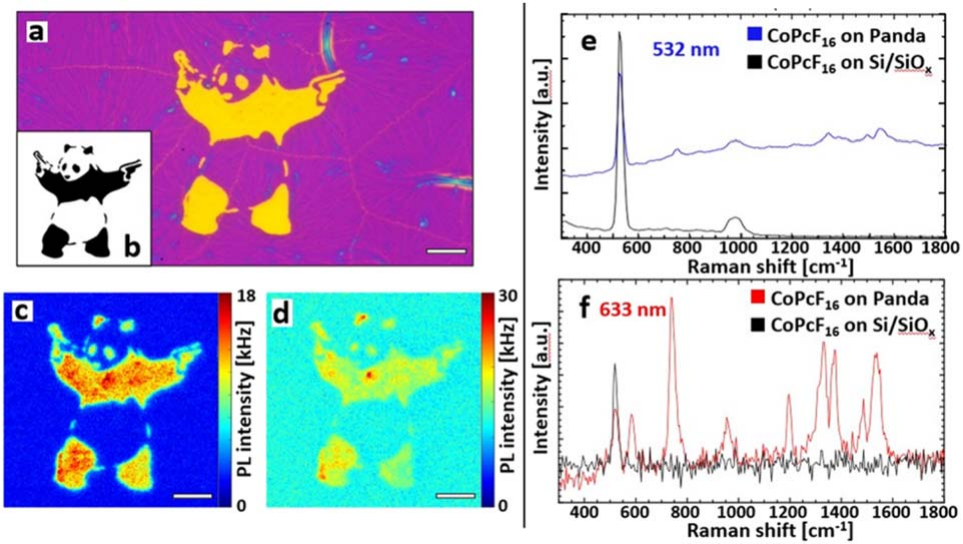
Optical investigations and Raman enhancement. (a) Optical micrograph of the Au-NS pattern on Si/SiO_*x*_. Scale bar: 10 μm. (b) Digital pattern that was used for (a). (c) PL image of the Au-NS-Panda covered with a submonolayer CoPcF_16_ illuminated with 532 nm (green light). Scale bar: 10 μm. (d) PL image of the same spot at excitation with 633 nm (red light) yields the CoPcF_16_ signal. Scale bar: 10 μm. (e) Spectra observed with 532 nm irradiation: the Raman bands of the CoPcF_16_ are residing on the PL of the Au nanospheres. (f) Typical enhanced CoPcF_16_ Raman spectrum taken inside the Au-NS-Panda with 633 nm excitation.

Under illumination with a wavelength of 633 nm, the luminescence image (Fig. 2a) shows three different areas, which can be distinguished by their brightness. The Si/SiO_*x*_-surface appears dark and shows only minor photoluminescence (PL) and almost no PL on the conventional gold electrodes (see Fig. 2a and black spectrum in Fig. 3f). In contrast to the other regions the laser written Au-NS pad clearly appears much brighter, where most of the signal intensity is caused by the CoPcF_16_ (see Fig. 3f, red spectrum). Fig. 2b–d display the SEM images of the pad, that were used to identify the nanospheres and to determine their size distribution. Naturally, most of the particles have spherical shapes with a few ones showing rod-like shapes and fused spheres. Their dimensions range from sub-10 nm to approx. 30 nm with an average size of 20.5 ± 7.2 nm and a maximal sphere-to-sphere distance of ≈50 nm. For the validation and measurement of the thickness of the evaporated CoPcF_16_ film grown on the substrate surface, we performed XPS with Al-Kα radiation (photon energy of 1486.6 eV). Applying the method by Seah and Dench,^50^ we utilized the intensity of the F 1s and Si 2s signals, considering their photoionization cross-sections,^51^ to assess the thickness of the molecular film. We observed a uniform sub-monolayer distribution of the molecules across the entire substrate.

### SERS enhancement on a free form pattered Au-NS substrate

With the described methods we fabricated a free form printed SERS substrate with the integrated pattern generator. Fig. 3a shows the optical micrograph of the Au-NS pattern that was used for the spectroscopic investigations. To illustrate the capabilities of the printing abilities we produced a pattern after a street art motive, the “Panda with Guns” (Fig. 3b), which will be further referred to as Au-NS-Panda. Again, a sub-monolayer CoPcF_16_ film was deposited on top of the whole substrate. Fig. 3c and d show the PL images obtained by excitation with 532 nm and 633 nm cw laser radiation, respectively.

Fig. 3e and f include the Raman spectroscopy analysis of the CoPcF_16_ molecule. The spectra were taken with an integration time of 10 ms and averaged over 10 frames. With an excitation at 532 nm (Fig. 3c) the Panda appears to have sharper features and the spectral intensity is dominated by a broad luminescence caused by PL emission of the Au nanospheres (Fig. 3e). The blue spectrum in Fig. 3a was taken from the surface of the Panda and shows the intense and broad PL signal from the Au-nanospheres and the Raman bands the CoPcF_16_ at 754 cm^−1^ and in the 1340–1550 cm^−1^ regime. The latter acts as a characteristic “fingerprint” for phthalocyanine molecules.^52^ The spectrum recorded on the Si/SiO_*x*_ substrate shows only the prominent Raman peak of Si/SiO_*x*_ at 520 nm and no Raman signal of the CoPcF_16_ is detected (Fig. 3d). However, by exciting the sample with a 633 nm laser, which is resonant to the LSPR of the Au-NS most of the signal is generated by surface enhanced Raman scattering (Fig. 3d) showing the distinctive vibrational bands of the CoPcF_16_ molecules and only a minor Pl background (Fig. 3e). The spectra in Fig. 3f reveal a detailed depiction of the CoPcF_16_ vibrational modes, as we obtain well-defined Raman peaks within a wide range. Additionally, to the Au-NS enhancement the excitation energy of 1.96 eV (633 nm) coincides with the HOMO–LUMO gap energy of the CoPcF_16_ (1.9 eV). The frequencies observed at 583, 742, 954, 1330, 1374, 1487 and 1538 cm^−1^ are in good accordance with literature,^53^ including reasonable shifts that derive from the influence of the fluorine atoms. The peaks at 1538, 1487, 954, 742, and 583 cm^−1^ can be assigned to the in-plane symmetric N=C stretching mode, benzene C–C stretch, macrocycle breathing, in-plane symmetric N-metal stretch and macrocycle breathing, respectively. For the repeated time, spectra observed outside the Au-NS-Panda just contain the silicon band at 520 cm^−1^.

We estimate our analytical Raman enhancement factor (AEF) as described by Le Ru *et al.*^14^
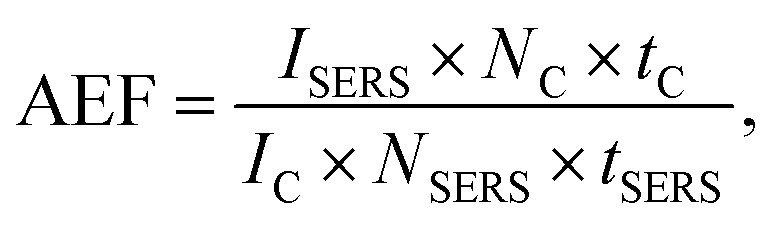


With (*I*_C,SERS_) as peak intensity, (*N*_C,SERS_) number of molecules responsible for the respective signal, (*t*_C,SERS_) integration time. Since no confocal Raman signal could be detected even after 5 minutes integration time, we take the noise level as an upper limit for the confocal Raman signal *I*_C_. As a further simplification we assume the number of molecules *N*_C_, equal to *N*_SERS_. Using for *I*_SERS_ the intensity the Raman peak at 742 cm^−1^, we calculate a minimum enhancement factor of 10^6^ for 633 nm and 10^4^ to 10^5^ for 532 nm. However, this is a very conservative approximation. Most likely the number of molecules within the hot spots will be much less than in the confocal focus and the intensity enhancement will be much higher.

## Materials and methods

### Materials

Silicon/silicon dioxide (Si/SiO_*x*_) wafer with 200 nm SiO_*x*_ layer and n-doped Si were purchased from Siegert Wafer. CoPcF_16_ was purchased from Sigma-Aldrich.

For the reaction solution 3 mmol (1304 g) of ^*n*^Bu_3_PAuCl was dissolved in 60 ml of ethanol and a suspension of 3 mmol (0.114 g) NaBH_4_ in ethanol was added. After stirring for 1 h the solvent was removed under reduced pressure. The residual black solid was extracted with dichloromethane and layered with three times the amount of diethyl ether. After 1 week a gold mirror formed leaving a dark supernatant. The dark brown supernatant was filtered and concentrated under vacuum. After the solution was stored at −30 °C for a few days, crystals of Au_32_(^*n*^Bu_3_P)_12_Cl_8_ formed (90 mg 12.6 μmol, 14%), that were used to prepare the ink.

### Printing and optical investigations

Au_32_ solutions (0.5 mM, 9 mg single crystalline Au_32_(^*n*^Bu_3_P)_12_Cl_8_ in 2 ml hexane) served as the ink for the thin film coating of the substrates, using a home-built spin-coater under ambient conditions. 200 μl ink was drop-casted on Si/SiO_*x*_ substrates, and after a short resting period, spin-coating was performed at a rotation speed of 780 rpm for 60 s.

Printing of the Au-NS patterns on Si/SiO_*x*_ was performed *via* raster scanning on a home-built confocal microscope driven by a controller and pattern generator (HydraSpex HydraLabX1) with a piezoelectric stage (Physik Instrumente P-517.3CL), by exposing the area of interest to 488 nm laser radiation (cw, iBeam-Smart diode laser, Toptica Photonics). The excitation beam was focused onto the substrate with an air objective lens (Carl Zeiss, NA = 0.70). The applied printing method included full laser intensity (1.86 mW), 0.003 s exposure time and three pattern iterations. The position accuracy of our writing process is given by our hardware and only limited by the closed loop scanning stage. This stage has a capacitive sensor feedback with a repeatable position accuracy of 10 nm. Hence, even with multiple iterations our patters have only a max. error of 10 nm. The dimension of our smallest possible structures that can be written determined by the diffraction limit of the used objective and sample material. On glass we can use a high NA oil objective and reach *λ*/2 with ∼250 nm with air objectives on *e.g.*, silicon the smallest patterns have sizes ∼300 nm, which has demonstrated in Geladari *et al.*^47^

Optical investigations were conducted using a home-built confocal microscope, equipped with a cw laser emitting radiation at 532 nm (100 μW for PL images and 22 mW for spectra, Coherent Sapphire) and at 633 nm (149 μW for PL images and 0.55 mW for spectra, Melles Griot 10-LHR-111). Photoluminescence images were acquired using an avalanche photodiode (APD, SPCM-AQR-14, PerkinElmer). For the SERS measurements, an UV-Vis-spectrometer (SP-2500i, Princeton Instruments) equipped with a charge-coupled device (CCD) camera (ProEM:512B+, Princeton Instruments) was used.

Deposition of CoPcF_16_ was done under ultra-high vacuum (UHV) conditions (base pressure of the deposition chamber 2 × 10^−8^ mbar) *via* molecular beam epitaxy with a home-build Knudsen cell. The molecules were placed in a crucible and deposited by resistively heating the cell to 390–400 °C. The temperature was adjusted till a constant deposition rate of 0.2–0.3 nm min^−1^ could be monitored by a quartz microbalance. XPS measurements were performed on a multichamber UHV system with a base pressure of 5 × 10^−10^ mbar. The analysis chamber is equipped with a monochromated Al Kα radiation source (XR 50 M, Specs) and a Phoibos 150 hemispherical photoelectron analyser (Specs). For calibration core-level peak positions of cleaned (Ar ion sputtering) gold (Au 4f_7/2_, 84 eV) and cupper (Cu 2p_3/2_) foils were used.

## Conclusions

In this work, we have introduced a new facile method for the generation of free form patterned SERS substrates with μm resolution. After structural characterization of the produced gold nanoparticles *via* SEM imaging, we used a sub-monolayer CoPcF_16_ film on Au-NS printed Si/SiO_*x*_, to discuss the occurring SERS performance and compare the influences of two different excitation wavelengths. We perceive much stronger Raman enhancement at 633 nm in comparison to 532 nm excitation, because of two phenomena: (1) the molecular resonance of the CoPcF_16_ and (2) the LSPR of the Au-NPs in the red spectral region. At illumination with 532 nm the predominant effect is the d-band luminescence of the Au itself. Considering the fact, that only a sub-monolayer of CoPcF_16_ was used, the characteristic molecular Raman signals can be detected simultaneously to a well-recognizable degree and the samples exhibits high SERS sensitivity. The presented laser-induced printing of gold nanospheres is a promising technique for the fabrication of SERS substrates at specific desired sample positions. It comprises low-cost production and simple applicability with high precision positioning and sensitivity. As an outlook, this method could be further optimized for a wide range of applications in chemical and biological analysis, for example in microfluidic channels or in portable analytical systems.

## Author contributions

O. G. developed and conducted the substrate inking and Au-NS printing, performed the photoluminescence, enhanced Raman and optical microscopy measurements. P. H. performed the CoPcF_16_ deposition and the XPS investigations. M. S. developed and synthesized the Au_32_ nanoclusters. A. M. performed the SEM measurements. M. E. and K. B. developed the pattern generator. O. G. and K. B. interpreted the results and wrote the manuscript. K. B., M. S., H. P., T. Ch., A. S. and A. J. M. conceived and supervised the project. All Authors have given approval to the final version of the manuscript.

## Conflicts of interest

There are no conflicts to declare.

## Supplementary Material
